# Consider *CUX1* variants in children with a variation of sex development: a case report and review of the literature

**DOI:** 10.1186/s12920-024-01945-0

**Published:** 2024-08-05

**Authors:** Lynn Tan, Shelley G. Young, Andrew H. Sinclair, Matthew F. Hunter, Katie L. Ayers

**Affiliations:** 1 Monash Genetics, Monash Health, Melbourne, VIC Australia; 2 Department of Paediatrics, Monash University, Melbourne, VIC Australia; 3 The Murdoch Children’s Research Institute, Melbourne, VIC Australia; 4 Department of Paediatrics, The University of Melbourne, Victoria, Australia

**Keywords:** CUX1, Hypospadias, Cryptorchidism, ASD, ADHD, Variation of sex characteristics, Disorder of sex development, Variation of sex development, VSD, DSD, Gonads, Testis

## Abstract

**Background:**

The *Cut Homeobox 1* (*CUX1*) gene has been implicated in a number of developmental processes and has recently emerged as an important cause of developmental delay and impaired intellectual development. Individuals with variants in *CUX1* have been described with a variety of co-morbidities including variations in sex development (VSD) although these features have not been closely documented.

**Case presentation:**

The proband is a 14-year-old male who presented with congenital complex hypospadias, neurodevelopmental differences, and subtle dysmorphism. A family history of neurodevelopmental differences and VSD was noted. Microarray testing and whole exome sequencing found the 46,XY proband had a large heterozygous in-frame deletion of exons 4–10 of the *CUX1* gene.

**Conclusions:**

Our review of the literature has revealed that variants in *CUX1* are associated with a range of VSD and suggest this gene should be considered in cases where a VSD is noted at birth, especially if there is a familial history of VSD and/or neurodevelopmental differences. Further work is required to fully investigate the role and regulation of *CUX1* in sex development.

## Background

The *CUX1* gene, previously known as *CUTL1* and *CCAAT displacement protein* (*CDP*), is evolutionarily conserved and universally expressed from *Drosophila melanogaster* to humans [1]. *CUX1* encodes two proteins (Cut-like homeobox 1, CUX1, and Cut alternatively spliced protein, CASP1) which arise from alternative splicing [2]. These proteins have different modes of activity, with the CUX1 isoform acting as a transcription factor and CASP1, which lacks the DNA binding motif of CUX1, acting as a Golgi protein [3]. *CUX1* regulates a myriad of developmental processes [4]. The CUX proteins are characterised by the presence of three CUT DNA binding domains in addition to a homeodomain (Fig. 2) [4]. Work in mouse models has shown that the *Cux1* gene plays an important role during the development of the upper layer of the brain cortex [5], and in humans variants in *CUX1* or its cis-regulatory regions have been associated with a variety of neurodevelopmental conditions or neurodiversity ranging from autism spectrum disorders (ASD) to developmental delay and intellectual disability [6, 7]. Animal models and cell culture have also revealed a role for *CUX1* in kidney development, cell migration and invasion and lung development (reviewed in [8]) and accordingly, patients with variants in *CUX1* can present with a variety of comorbidities ranging from craniofacial abnormalities, heart defects, lung defects to reproductive conditions such as disorders/differences/variations of sex development (VSD) [7]. VSDs are congenital conditions where sexual development is atypical. VSDs are often noted at or even prior to birth and can be the first indication of a broader syndrome, triggering a genetic investigation. Very little is known about *CUX1* in reproductive development. Both *Cux1* and *Cux2* are widely expressed in urogenital tissues in mice [9] where research suggests that *Cux1* may play a role in gonadal development, testosterone production and spermatogenesis [10–12]. A better understanding of the contribution of variants in *CUX1* to VSD and related conditions is required and may reveal the underlying role of *CUX1* in human reproductive development and health.

The role of *CUX1* in human sexual development and function has not been well reported. Here we present a new familial case where the proband who has a VSD (congenital complex hypospadias), neurodevelopmental differences, and subtle dysmorphism carries a multi-exonic deletion in the *CUX1* gene inherited from an affected mother. We have reviewed published clinical cases of *CUX1* variants to find individuals with a VSD or reproductive condition. This reveals a spectrum of VSD associated with *CUX1* variants, highlighting the importance of CUX1 in human reproductive development and its consideration as a diagnostic gene for children born with a VSD.

## Case presentation

The proband is a 14-year-old male who had congenital complex hypospadias, with torsion of the penis, chordee, penoscrotal tethering and a bifid glans within a glandular hypospadias. A MAGPI (meatal advancement and glanuloplasty incorporated) repair was undertaken at age 13 months. A right-sided hydrocele and left-sided cryptorchidism was also corrected. A right inguinal hernia repair was undertaken at age 23 months. Hypospadias revision surgery was undertaken with a degloving Nesbit plication and circumcision undertaken at age 11 years. Good surgical outcomes have not necessitated ongoing urology follow up at this time.

From a neurodevelopmental perspective, expressive and receptive language difficulties were noted on speech pathology assessment at age 5. Autism spectrum disorder (ASD) was diagnosed on formal psychological assessment age 6. This assessment also noted below average cognitive skills, significant working memory deficits, oppositional defiant behaviour and anxiety. Fine motor skill issues were also noted by the proband’s parents, with ongoing difficulties with movements like pen holding and footwear fitting. Previous hearing and vision assessment have been unremarkable. A full-scale intelligence quotient (IQ) assessment or cerebral imaging have not previously been undertaken. The proband currently attends a mainstream secondary school with a full-time aid. Dental enamel issues have necessitated removal of five teeth. Other past medical history includes asthma, peanut anaphylaxis and food allergies. An electroencephalogram (EEG) pursued at age 3 for single febrile convulsion was unremarkable.

The proband is the first child to non-consanguineous parents of European ancestry. No antenatal issues were reported. The proband was born at term with physical measurements ranging 22nd-64th percentile (head circumference 34cm, length 50cm, and weight 3.72kg). Phototherapy was administered for management of jaundice postnatally.

**Fig. 1:**
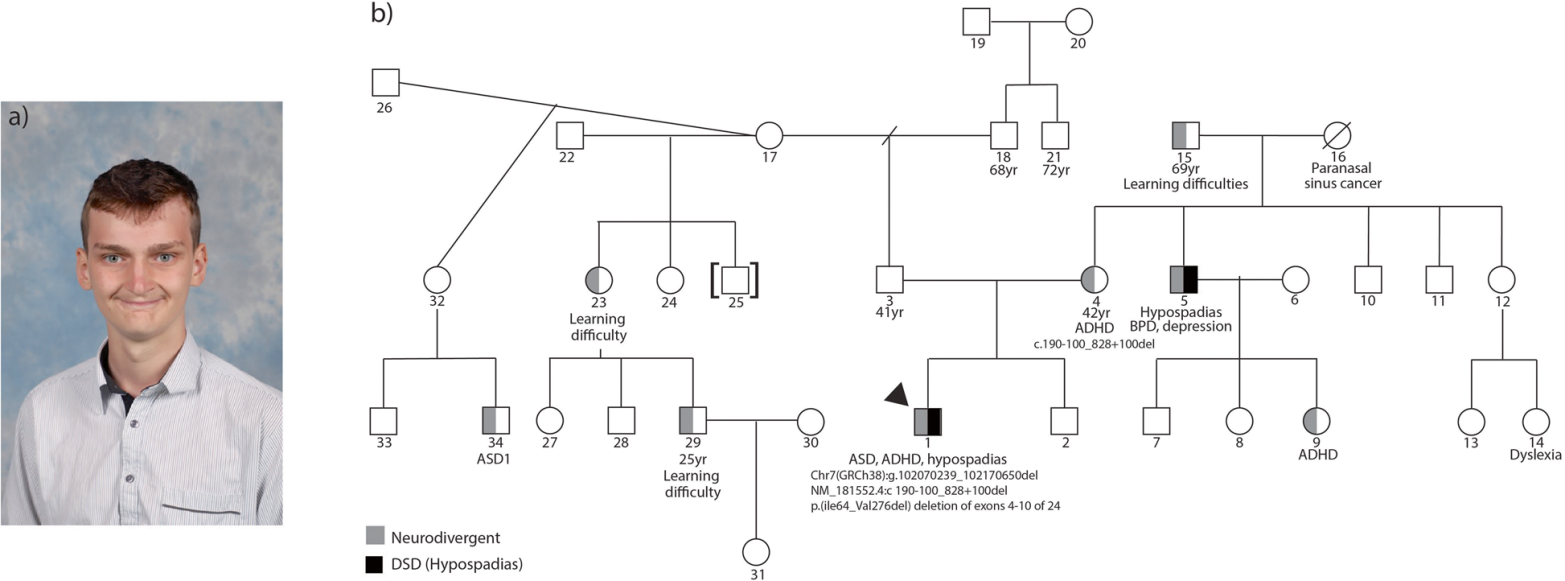
A novel familial variant in *CUX1* associated with VSD and neurodevelopmental differences. **a** picture of the proband highlighting facial features **b** pedigree illustrating the proband (arrow), the maternally inherited *CUX1* variant (4), maternal uncle with hypospadias who has not undergone segregation testing (5), and neurodevelopmental differences on both sides (denoted by the grey shading)

The proband’s physical measurements were 90-98th percentile (head circumference 58cm, height 178.5cm, and weight 73.8kg) at age 13. Retrognathia, a large nose, prominent supraorbital ridges with horizontal eyebrows, short philtrum, thin upper lip vermillion, and prominent digit pads were noted (Fig. 1). An approximately 10cm café au lait macule on the left lower back, as well as striae along the back and hips were also present. Other features were thought to be familial.

A family history of both neurodevelopmental differences and genital variations was revealed upon interview of the proband’s parents (Fig. 1). The proband’s mother has a diagnosis of ADHD (attention deficit hyperactivity disorder) and is awaiting assessment for ASD. The proband’s maternal uncle has a history of hypospadias. A female cousin (daughter of aforementioned uncle) also has a diagnosis of ADHD. The proband’s maternal grandfather experienced learning difficulties. The proband’s paternal half-aunt and paternal half-cousin also experienced learning difficulties with another male paternal half-cousin receiving a diagnosis of Level 1 ASD (Fig. 1). There are no known seizures, cerebral imaging abnormalities, congenital cardiac, or issues relating to dental enamel on either side of the family. No direct evaluation of the family was undertaken. No genetic testing has been undertaken in the larger family.

**Fig. 2:**
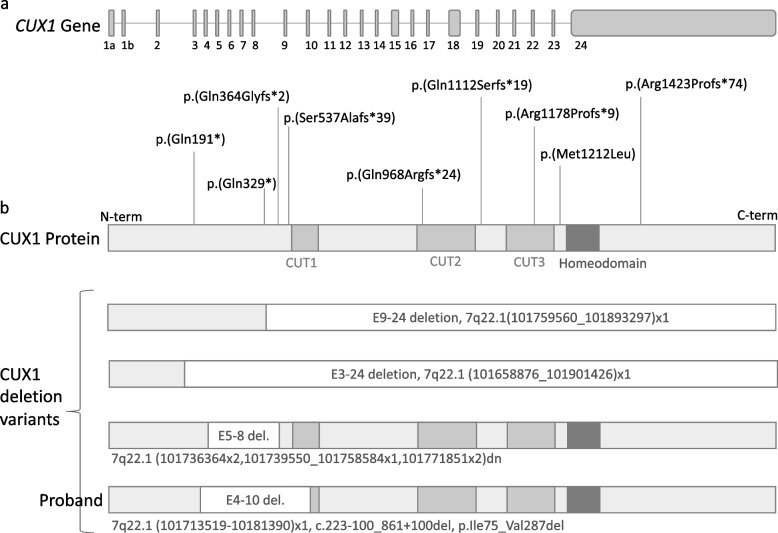
Genetic variants in the *CUX1* gene described in patients with a VSD from the literature and the current study. **a** Schematic of *CUX1* gene (adapted from [13]) **b** Schematic of variant CUX1 p200 isoform proteins of VSD patients from the literature and the proband (adapted from [7]). Missense and nonsense variants are highlighted along the CUX1 protein schematic and deletion variants are below the protein schematic

DNA molecular karyotyping and exome sequencing were undertaken as the current standard of care for evaluation of congenital abnormalities at our service. Molecular karyotyping on DNA from a saliva sample from the proband (Illumina Infinium GSA-24 v3.0 0.2Mb resolution) revealed a 46,XY karyotype and a large heterozygous in-frame deletion of exons 4–10 of the 24 exon *CUX1* gene. Interpretation is based on the UCSC GRCh37/hg19 human reference sequence. Trio whole exome sequencing was also undertaken which orthogonally confirmed the deletion: Chr7(GRCh38): g.102070239_102170650del, NM_001202543.1 c.223-100_861 + 100del, p.(Ile75_Val287del) (Fig. 2, Table 1). Library preparation was performed using a TWIST BioScience Library Preparation EF KIT (TWIST-Alliance) kit, with libraries sequenced on an Illumina NovaSeq6000. Data filtering was performed on 21 April 2023 using an in-house Genomics Annotation and Interpretation Application pipeline. Percent coverage of 97.69 at > 20 × was achieved at a uniformity of 97.96 for the proband exome. The data analysis pipeline is based on Gemini v18 with annotation from Ensembl Variant Effect Predictor (VEP) and dbNSPF. Copy number variants are identified by three CNV callers CoNIFER, DECoN and XHMM, and filtered according to morbid gene content relevant to the referral phenotype. Variants are reported according to HGVS nomenclature and classified according to the joint consensus recommendations from the American College of Medical Genetics and Genomics and the Association for Molecular Pathology. This variant was classified by the reporting laboratory as likely pathogenic and shown to be maternally inherited. No other variants of interest were reported on the trio whole exome sequencing. Fragile X testing was unremarkable.

**Table 1:** *CUX1* variants in patients with a reported variation of sex development

Patient	Reference (patient)	Gender	Genomic test	Mutation type	Affected protein	Variant details				Inheritance	Variation of sex development
						**Genomic change (hg19)**	**Genomic change (hg38)**	**Transcript change (NM_001202543.2)**	**Protein change (NP_001189472.1)**		
**1**	Oppermann et al. (4)	Male	Trio WES	Nonsense	CUX1, CASP	Chr7:101754985C > T	Chr7:102111705C > T	c.571C > T	p.Gln191*	de novo	Redundant foreskin
**2**	Oppermann et al. (6)	Male	Trio WES	Nonsense	CUX1, CASP	Chr7:101821872G > T	Chr7:102178592G > T	c.985G > T	p.Glu329*	maternal	Unilateral cryptorchidism
**3**	Oppermann et al. (8)	Male	Singleton WES	Deletion	CUX1, CASP	Chr7:101833133del	Chr7:102189853del	c.1091del	p.Glu364Glyfs*2	paternal	Penile hypospadias, bilateral cryptorchidism
**4**	Oppermann et al. (11)	Male	Singleton WES	Deletion	CUX1	Chr7:101840267del	Chr7:102196987del	c.1609del	p.Ser537Alafs*39	de novo	Bilateral cryptorchidism
**5**	Oppermann et al. (17)	Male	Singleton WES	Frameshift	CUX1	Chr7:101845479del	Chr7:102202199del	c.2935del	p.Glu979Argfs*24	paternal	Bilateral cryptorchidism
**6**	Oppermann et al. (21)	Male	Trio WES	Deletion	CUX1	Chr7:101870817del	Chr7:102227537del	c.3334del	p.Gln1112Serfs*19	de novo	Micropenis
**7**	Oppermann et al. (22)	Male	Trio WES	Deletion	CUX1	Chr7:101877398_101877401del	Chr7:102234118_102234121del	c.3533_3536del	p.Arg1178Profs*9	maternal	Bilateral inguinal hernia, penile adhesions
**8**	Oppermann et al. (24)	Male	Trio WES	Missense	CUX1	Chr7:101877499A > T	Chr7:102234219 > T	c.3634A > T	p.Met1212Leu	de novo	Cryptorchidism
**9**	Oppermann et al. (31) Platzer et al. (9)	Male	Microarray	Large deletion	CUX1, CASP	arr[GRCh37] 7q22.1(101658876_101901426) × 1		del exons 3–24	p.?	de novo, mosaic	Hypospadias
**10**	Oppermann et al. (32) Platzer et al. (8)	Male	Microarray	Large deletion	CUX1, CASP	arr[GRCh37] 7q22.1(101759560_101893297) × 1		del exons 9–24	p.?	de novo	Hypospadias
**11**	Oppermann et al. (F1)	Female	Microarray	Deletion	CUX1	arr[GRCh37] 7q22.1(101736364 × 2,101739550_101758584 × 1101771851 × 2)dn		del exon 5–8	p.?	de novo	Normal external genitalia but hemi-uterus
**12**	Oppermann et al. (F2)	Female	Trio WGS	Frameshift	CUX1	Chr7:101892035_101892036dup	Chr7:102248755-102248756dup	c.4264_4265dup	p.Ala1423Profs*74	de novo	Clitoris hypertrophy
**13**	This report	Male (XY)	Microarray/Trio WES	Deletion	CUX1	Chr7:101713519_101813930del	Chr7:102070239_102170650del	c.223-100_861 + 100del	p.Ile75_Val287del	maternal	Torsion of the penis, chordee, penoscrotal tethering and a bifid glans within a glandular hypospadias

Variant co-ordinates are provided in hg19 and hg38

*WES* whole exome sequencing

### Review of the literature and spectrum of VSDs associated with *CUX1* variants

In humans, variants in *CUX1* have been associated with a wide variety of phenotypes including intellectual disability and developmental delay, delayed speech or language development, MRI abnormalities and heart defects (OMIM:618330) [6, 7]. Other phenotypes include facial dysmorphologies, short stature and seizures. Our literature review revealed just 12 cases from two reports in which VSD or related reproductive conditions were described in individuals with *CUX1* variants (Table 1). This includes a recent report in which Oppermann and colleagues collated clinical data on 34 individuals with a range of missense and null variants in *CUX1* including 23 new patients and 11 previously reported [6, 7]. Among the reported phenotypic abnormalities of the body and face, abnormality of the male genitalia was overall the most commonly reported feature, exceeding neurological and CNS features or specific individual neurodevelopmental phenotypes [7]. Indeed, 63% of male individuals in this publication (10 of 16) had a reported VSD/abnormality of the male reproductive organs. This was as high as 69% in those with null variants (9 of 13). Abnormalities of the male reproductive organs described include micropenis, hypospadias, cryptorchidism, and defects of the foreskin (Table 1). These individuals had a variety of *CUX1* variants with variants likely to cause a loss of function such as nonsense and frameshift variants or multi-exonic deletions most common in those with a VSD (11 of 12 patients). Just one patient with a VSD had a missense variant which lies between the third CUT domain and the homeobox (Table 1, Fig. 2). In addition to our patient, multi-exonic deletions have been described in two male patients with hypospadias previously. Although unlike in our case, these deletions were de novo. In addition, the researchers described two female individuals with *CUX1* variants and a VSD (one with clitoris hypotrophy and another with reported hemi-uterus). Both had damaging variants i.e. multi-exonic deletions or frameshift changes (Table 1).

## Discussion and conclusions

*CUX1* variants are associated with a syndrome that includes a range of phenotypes [7]. The predominantly recognised phenotype is that of a neurodevelopmental spectrum, with variable neurological, CNS and distinctive facial and physical differences. Of physical differences associated with *CUX1* variants, abnormalities in sex organ development are commonly present, particularly in males [6, 7].

This report is the third to associate a *CUX1* variant to this syndromic condition. Our proband’s neurological presentation falls within the known spectrum associated with *CUX1* variants. This report adds importantly highlights the VSD phenotype of this condition, which is less well recorded in the literature. As a VSD is often noted at or before birth, it can be the first indication of a broader syndrome. Thus, it is imperative that we understand the contribution of *CUX1* variants to VSD, as a genetic diagnosis of a *CUX1*-related condition may trigger cascade testing in relatives or allow the family to seek early intervention, and pre-emptively screen and monitor for associated features such as ASD, learning difficulties, or heart defects. Furthermore, in some *CUX1* cases, developmental delay and intellectual disability improves with age [6, 7] thus a *CUX1* diagnosis may inform families of a better prognosis. Better knowledge of *CUX1* related VSDs may allow families to understand recurrence risks and to potentially seek assisted reproduction methods.

The reproductive features reported in individuals with *CUX1* variants vary, with the underlying molecular mechanisms as yet unclear. As reviewed here, in males, associated features include micropenis, hypospadias, cryptorchidism and defects of the foreskin [6, 7]. These features could be caused by lower-than-normal androgens or hypogonadism. Murine models are consistent with this. In mice, *Cux1* expression has been documented in testis, specifically in the Sertoli cells and spermatids during spermatogenesis [10]. Mice homozygous for a c-terminal deletion of important protein domains including the third CUT repeat and homeobox domain exhibit significantly reduced fertility, rarely producing offspring. Although testes were grossly normal in appearance in these males, both the homozygous mutant and heterozygote (fertile) mice had significantly lower serum testosterone levels when compared the wild-type mice [11]. CUX1 exists as multiple isoforms that arise from proteolytic processing of a 200-kDa protein or an alternate splicing or from the use of an alternate promoter. The 200-kDa protein has a role in cell proliferation. Transgenic mice constitutively overexpressing this 200-kDa CUX1 protein have larger testes and higher testosterone levels, proposed to be due to an increase in the number of hormone-producing Leydig cells [10]. In patients with *CUX1* variants, reduced testosterone levels is yet to be confirmed. Reduced testosterone can be caused by primary hypogonadism (a defect in the testes) or secondary hypogonadism (hypogonadotrophic hypogonadism), where defects in the hypothalamus or pituitary gland lead to reduced gonadotrophins which are important to stimulate testicular testosterone production. Indeed in in vitro assays, CUX1 has been shown to regulate Kisspeptin [14], a principal activator of gonadotrophin releasing neurons, whose loss can cause hypogonadotrophic hypogonadism. The KISS1 system is a prerequisite for the onset of puberty and maintenance of normal reproductive function. Additionally, Sertoli cell *Cux1* expression is continuous in prepubertal mice, and becomes phasic when spermatids are first present at postnatal day 21. *Cux1* transgenic mice constitutively overexpressing the 200-kDa CUX1 protein did not switch to asynchronous expression until postnatal day 28, suggesting that forced expression of the 200-kDa CUX1 protein appeared to slow the initial spermatogenic cycle [10]. In contrast, a testis specific 55 kDa isoform has been described to be abundant in round spermatids [12]. As CUX1 was not expressed in proliferating cells in testes from wild-type or transgenic mice constitutively overexpressing the 200-kDa CUX1 protein, this may indicate that the testis-specific form of CUX1 is not involved in cell division and but rather in signalling between the developing germ cells and their supporting Sertoli cells [10]. In summary, CUX1 involvement in male reproductive development could be multi-pronged and may explain why 46,XY patients with *CUX1* variations exhibit a range of VSD. Endocrinological and gonadal investigations are required in affected individuals to determine the underlying cause of VSD.

The role of *CUX1* in female development is even less clear, and reproductive features may be underreported in female patients. In the study by Opperman et al., one female individual reported with clitoral hypertrophy, and another reported with a hemi-uterus [7]. Of note, while these individuals were reported as female, confirmation that individuals have a female genetic sex (46,XX) (i.e. there is no sex reversal) is required as both clitoral hypertrophy and uterus defects have been reported in 46,XY females with gonadal dysgenesis [15, 16]. It is possible however that CUX1 also plays a direct role in female reproductive development too. In human fetal gonad transcriptomic datasets *CUX1* is expressed across most cell types in both testes and ovaries [17]. In adult human transcriptomics data the uterus is the tissues with the highest *CUX1* expression [18]. In both male and females with *CUX1* variants, endocrinology and fertility testing may shed further light on the exact underlying cause of the VSD presentation. Further work is also needed to define the range of VSDs in females.

Finally, it is important to note that both incomplete penetrance and variable expressivity are associated with *CUX1* variants. Our report is also the first describing a familial inheritance of a multi-exonic deletion associated with a VSD. The variant is inherited from an affected mother, with individuals on the maternal side of the family presenting with VSD or neurodevelopmental differences. However, it is interesting to note that the paternal extended family also reported neurodevelopmental features highlighting the possibility that oligo/multigenic factors may contribute, although no additional diagnostic findings were reported from WES analysis. Further research into the genetic and environmental factors that influence CUX1-associated phenotypic presentation is needed.

In summary, we describe a patient with complex hypospadias, neurodevelopmental differences and subtle dysmorphism who has a familial deletion variant in the *CUX1* gene. As the third report to describe variants in *CUX1* associated with a VSD, it solidifies the connection between this gene and sex development processes. We suggest that *CUX1* variants be considered in patients presenting to genetic clinics with a VSD phenotype, especially in cases where there is a family history of neurodiversity or other associated conditions.

## Data Availability

Permission for use of images/Material credits -Images within this article (including proband image and pedigree) are excluded from the Creative Commons Licence and permission to reproduce them anywhere else is required. Genomic data is not available due to privacy and ethical constraints.

## Abbreviations

VSD: Variation of sex development
ASD: Autism spectrum disorder
CNS: Central nervous system
WES: Whole exome sequencing
